# CSSF-CLIP-HSQMBC: measurement of heteronuclear coupling constants in severely crowded spectral regions†

**DOI:** 10.1039/c9ra04118d

**Published:** 2019-11-05

**Authors:** Aitor Moreno, Kine Østnes Hansen, Johan Isaksson

**Affiliations:** Bruker BioSpin AG, Application Science Department CH-8117 Fällanden Switzerland; Marbio, UiT – The Arctic University of Norway Breivika NO-9037 Tromsø Norway; Department of Chemistry, UiT – The Arctic University of Norway Breivika NO-9037 Tromsø Norway johan.isaksson@uit.no

## Abstract

A new pulse program development, a chemical shift selective filtration clean in-phase HSQMBC (CSSF-CLIP-HSQMBC), is presented for the user-friendly measurement of long-range heteronuclear coupling constants in severely crowded spectral regions. The introduction of the chemical shift selective filter makes the experiment extremely efficient at resolving overlapped multiplets and produces a clean selective CLIP-HSQMBC spectrum, in which the desired coupling constants can easily be measured as an extra proton–carbon splitting in f2. The pulse sequence is also provided as a real-time homonuclear decoupled version in which the heteronuclear coupling constant can be directly measured as the peak splitting in f2. The same principle is readily applicable to IPAP and AP versions of the same sequence as well as the optional TOCSY transfer, or in principle to any other selective heteronuclear experiment that relies on a clean ^1^H multiplet.

## Abstract 2

A new pulse program development, a chemical shift selective filtration clean in-phase HSQMBC (CSSF-CLIP-HSQMBC), is presented for the user-friendly measurement of long-range heteronuclear coupling constants in severely crowded spectral regions.

## Graphical abstract

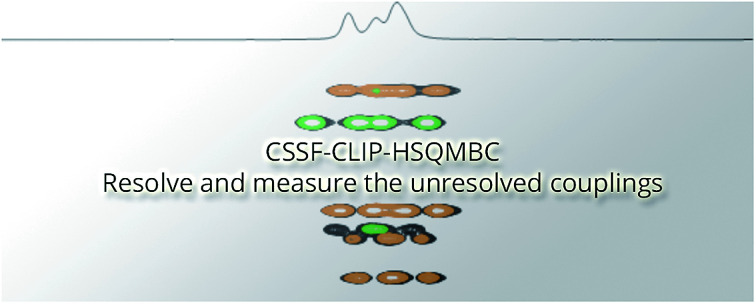

## Introduction

Long-range heteronuclear scalar couplings contain important information about molecular relative configuration, structure identity and structural conformation.^1–4^ The size of these couplings are in the same range as proton–proton scalar couplings, 0–15 Hz, but are generally more complicated to measure accurately. There is a large number of different pulse sequences available for the measurement of long range heteronuclear scalar couplings, mainly divided into TOCSY based- and HMBC/HSQMBC-based methods. The pros and cons with these have been comprehensively reviewed.^5–7^ In short, no technique has so far proven to be a generally applicable method to measure long range couplings, but it is always a matter of choosing the most appropriate technique with regards to the amount of sample, the sample complexity (overlap), whether the carbon is protonated or not, and how many couplings need to be measured simultaneously.

A user-friendly approach for measuring long range proton–carbon scalar couplings has been proposed by Saurí *et al.*^8^ The CLIP-HSQMBC uses a selective pulse on a proton signal to remove interfering homonuclear couplings. This is crucial to assure pure absorptive lineshapes to allow the accurate measurement of coupling constants. The HSQMBC then produces carbon coupled in-phase multiplets in which the relevant proton–carbon coupling produces an extra splitting in f2 compared to the corresponding proton multiplet in an ordinary proton 1D spectrum.

Severely crowded spectral regions present a challenge for the measurement of coupling constants in general. Spectral overlap in both the proton and carbon dimensions of non-homonuclear coupled protons is not uncommon in complex molecules *e.g.* for (a) multiple residues of the same amino acid in modified peptides, (b) (deoxy)-ribose moieties in nucleic acids, (c) carbohydrates, (d) pseudo-symmetric parts of small molecules (see securidine A example below), or (e) in stretches of repeated atoms like for example in molecules containing (partly unsaturated) lipids. Another case is coincidentally overlapping proton signals coupling to the same carbon through long-range scalar coupling. This can arise for example in natural products containing many aliphatic protons like modified cyclic peptides, polyketides, macrolides, steroids, saponins, terpenoids, glycosides *etc.* Difficulties with spectral overlap can sometimes be circumvented by coupling the experiment to a TOCSY element, which situationally allows the selection of one out of several overlapping resonances.^8^ A 3D HSQC-HSQMBC approach has also been proposed to address the problem of spectral overlap,^9^ as well as a *J*-scaled CLIP-HSQMBC.^10^

In order to overcome this limitation we here propose to apply gradient enhanced proton chemical shift selective filtration (CSSF)^11^ as the selection element in the CLIP-HSQMBC method. The ability to very cleanly select an unresolved multiplet in the proton dimension also results in a reduced number of observed correlations in the carbon dimension, thereby reducing the risk of inconvenient overlaps, both direct and folded/aliased, within the sampled carbon spectral width. We have denoted the pulse sequence development: CSSF-CLIP-HSQMBC.

An important limitation to get straightforward coupling measurements directly from in-phase separated peaks in the parent CLIP-HSQMBC is the requirement that no other proton coupled with the proton of interest may be excited by the selective pulse, as this will add dispersive contributions to the lineshape. It should be noted that this limitation is not overcome by the chemical shift selective filter even though it visually appears entirely clean. It is only through the selectivity of the shaped pulse that contributions from *J*_HH_ couplings can be avoided, whereas the CSSF cleans up any off-resonance chemical shifts that are excited by the selective pulse.

It is highly attractive to simplify the multiplet pattern of the crosspeaks, which results from the homo- and heteronuclear couplings, to a simple doublet in f2 split by the heteronuclear coupling constant. In the case where there is no spectral overlap, a PSYCHE version of HSQMBC to achieve spectrum-wide homonuclear decoupling has been reported.^12^ In order to achieve homodecoupling in the CSSF-CLIP-HSQMBC experiment, a version with real-time band-selective homodecoupling (bshd) during acquisition has been prepared.^13,14^ We demonstrate that even though coupling constant measurement in the direct dimension of homodecoupled spectra can be treacherous, the heteronuclear coupling constants can be reliably measured directly as the splitting of the doublet in f2. This is possible as long as certain experiment conditions were scaling occurs are avoided.

## Results and discussion

The new pulse sequence development, the CSSF-CLIP-HSQMBC, makes use of a chemical shift selective filter as the means to achieve a clean in-phase selection of the proton of interest.^11^ CSSF is an iterative method that adds up the on-resonance signal while off-resonance contributions are eliminated by destructive averaging because of differences in chemical shift evolution. The co-addition of FIDs makes this method extremely selective and allows the measurement of scalar couplings that may otherwise have been considered unmeasurable because of severe spectral overlap. Successful selection only requires a spectral separation of 1–2 Hz in the proton resonance frequency, and is thus able to resolve multiplets that appear to coincide.

**Fig. 1:**
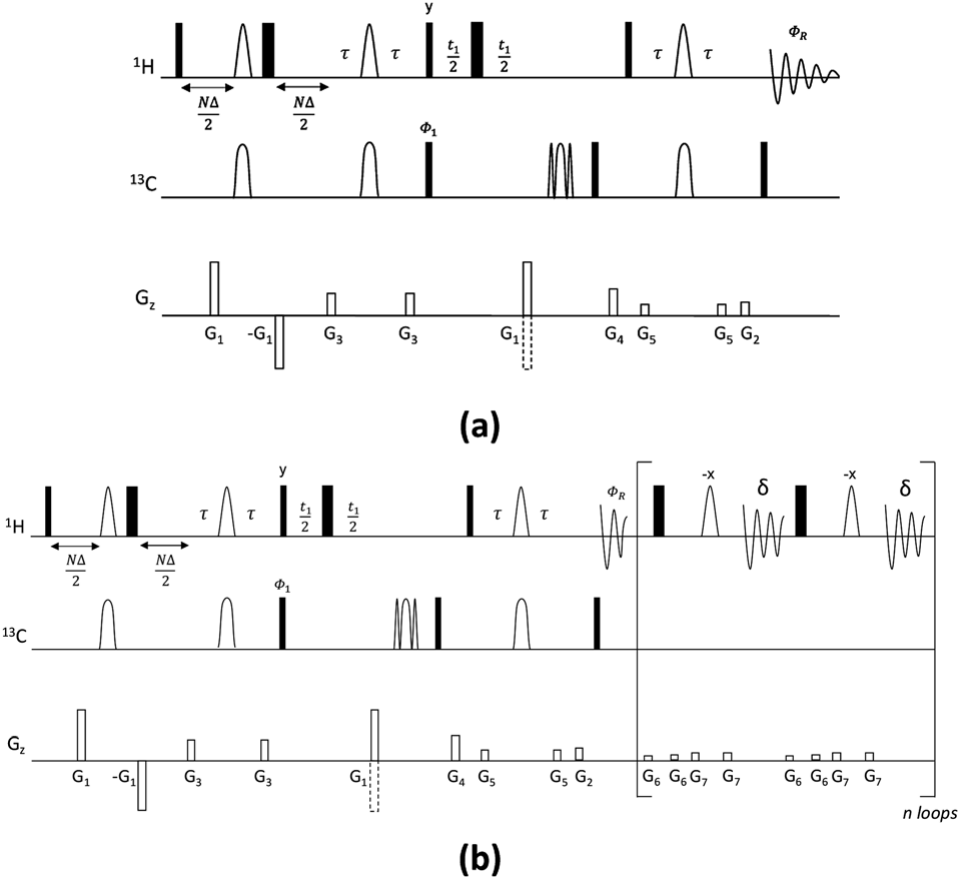
Pulse sequences for the CSSF-CLIP-HSQMBC (a) and CSSF-CLIP-HSQMBC_bshd (b) experiments. Narrow and wide rectangles represent 90° and 180° pulses, respectively, applied from the *x* axis unless otherwise specified. Unfilled arcs on ^1^H represent 180° selective refocusing pulses, while unfilled arcs on ^13^C represent 180° adiabatic inversion and refocusing pulses, respectively. *D* is the increment of the CSSF, *N* = 0, 1, 2…*n*, where *t*_max_ = (*n* + 1)*D*. The delay *τ*′ (=1/(4^*n*^*J*_CH_) = *τ* + *p*180/2, where *p*180 is the duration of the selective 180° ^1^H pulse) is an INEPT transfer delay. A minimum two-step phase cycle is applied: *Φ*_1_ = *Φ*_R_ = *x*, −*x*. Gradients *G*_1_ and *G*_2_ are used for coherence selection using the echo-antiecho protocol, *G*_4_ acts as a zz-filter, and *G*_1_, *G*_3_, *G*_5,_*G*_6_ and *G*_7_ flank the selective refocusing proton pulses and hard 180° pulses, respectively. The following pulsed field gradients were used: *G*_1_ = 40 G cm^−1^, *G*_2_ = 10 G cm^−1^, *G*_3_ = 17 G cm^−1^, *G*_4_ = 25 G cm^−1^, *G*_5_ = 9 G cm^−1^, *G*_6_ = 1.5 G cm^−1^, *G*_7_ = 2.5 G cm^−1^. For the CSSF-CLIP-HSQMBC_bshd experiment (b) homonuclear decoupling during the acquisition time (AQ) is performed using refocusing blocks including a pair of hard and selective 180° 1H pulses applied at intervals of 2*δ* = AQ/*n*, where *n* is the number of loops.

The CSSF-CLIP-HSQMBC sequence shown in Fig. 1 contains two modules: the gradient-selected (gs)-CSSF^11^ and a CLIP-HSQMBC^8^ sequence, followed by ^1^H detection in the absence of ^13^C decoupling. The first part of the pulse sequence is the gs-CSS filter, which allows the highly selective excitation of overlapping proton signals of interest with a reported resolution of up to 1.4 Hz. The selectivity of the CSSF is given by Δ*ν* = 0.5/*t*_max_, where *t*_max_ is the maximum chemical shift evolution interval and Δ*ν* is the chemical shift difference between the overlapping protons. The second part is a CLIP-HSQMBC sequence that allows the clean observation of ^13^C-isotopomer signals in which both the heteronuclear and homonuclear couplings exhibit pure in-phase character. This is achieved by the selective 180° proton pulses which eliminate homonuclear coupling modulations and hence avoid signal distortions. Further, heteronuclear antiphase components are eliminated by the application of a 90° carbon pulse before acquisition resulting in pure absorptive lineshapes. In order to speed up the acquisition, non-uniform sampling can readily be applied in the indirect dimension. The full pulse sequence is attached in the ESI.†

### Quinine

**Fig. 2:**
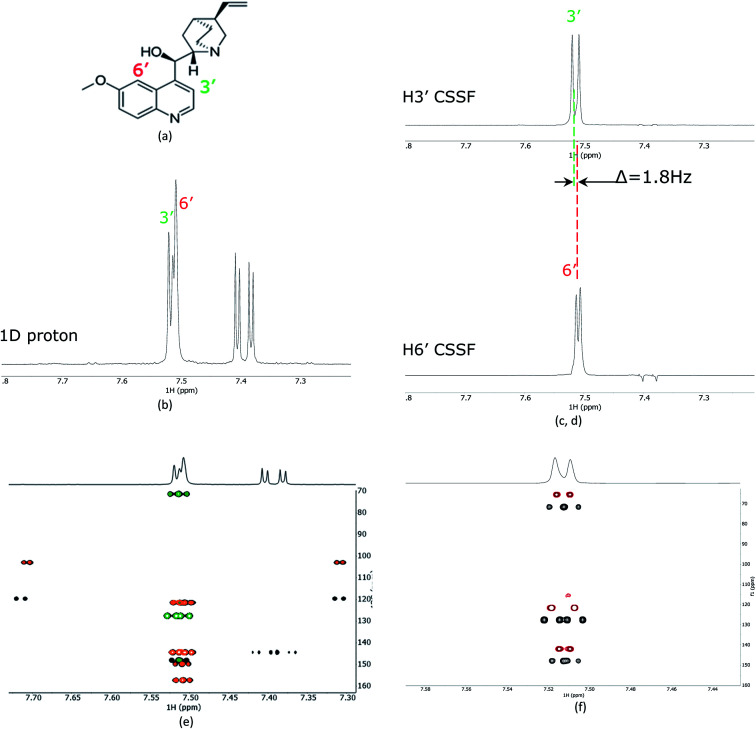
(a) The structure of quinine, (b) 1D proton spectrum showing the partially overlapping H3′ and H6′ doublets, (c) the CSSF selected (selcssfzg) clean doublets of H3′ and (d) H6′. (e) Superimposed selective CLIP-HSQMBC (black), with CSSF-CLIP-HSQMBC of H3′ (green) and H6′ (orange) using the corresponding CSSF setting applied in (c) and (d). In (f) the H3′ selected CSSF-CLIP-HSQMBC (black) is superimposed with an offset on the real-time bshd CSSF-CLIP-HSQMBC (red).

As a proof of principle, the CSSF-CLIP-HSQMBC was compared to the parent CLIP-HSQMBC experiment using a sample of 50 mM quinine in DMSO-*d*_6_ (Fig. 2). Even though the H3′ and H6′ protons are only separated by 1.8 Hz in a proton 1D spectrum acquired at 400 MHz proton frequency, the CSSF allows the selection of the near perfectly clean individual doublets using eight added FIDs (*t*_d0_ = 8) with different chemical shift evolution periods, effectively removing all off-resonance contributions (Fig. 2c and d). The clean doublets display the couplings ^3^*J*_H3′H2′_ = 4.5 Hz and ^4^*J*_H6′H8′_ = 2.8 Hz. The corresponding CSSF-CLIP-HSQMBC produces clean 2D spectra consisting only of correlations originating at the selected proton and the observed splitting patterns are in this case identical to the original CLIP-HSQMBC sequence (Fig. 2e) as none of the carbons simultaneously couple strongly enough with both of the two overlapping protons (H3′ and H6′).

The multiple-bonds proton–carbon couplings can easily be measured as an extra splitting in the respective HSQMBC crosspeak. Both reference peaks are doublets and the CLIP-HSQMBC peaks are resolved doublets of doublets.

The splittings due to homonuclear couplings in f2 in the CSSF-CLIP-HSQMBC spectrum can be eliminated by applying a real-time band-selective homonuclear decoupling scheme during acquisition (pulse sequence in Fig. 1b, and spectra in Fig. 2f). This is not as uncomplicated as it might first appear as pulsing during windowed acquisition is known to be able to cause *J*-scaling, phase shifts and chemical shift shifts.^15–19^ We do however show empirically that, as long as the length of the acquisition blocks are approximately twice as long as the selective pulse, no detectable scaling occurs and the method is robust for measuring long-range heteronuclear couplings in the direct dimension, under real-time band-selective homonuclear decoupling conditions (Fig. S1–S4 in the ESI†).

### Securidine A

**Fig. 3:**
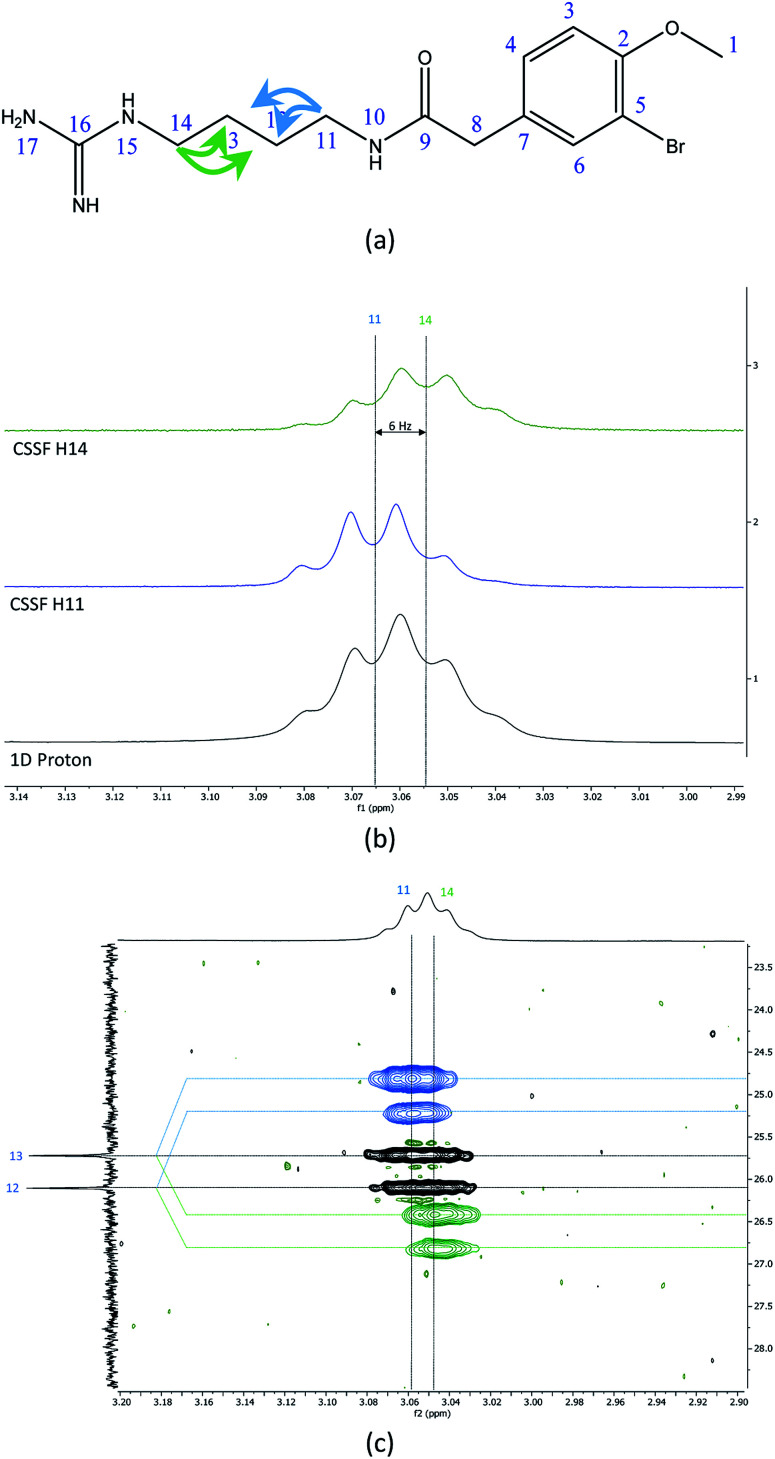
(a) The chemical structure of securidine A, (b) stacked plots of the 1D proton spectrum of the overlapped H11 and H14 multiplets (bottom), with the cleanly filtered H11 (middle) and H14 (top) quartets. (c) The CSSF-CLIP-HSQMBC crosspeaks of the ^2^*J*_H11C12_ and ^3^*J*_H11C13_ (blue, offset above) as well as the ^2^*J*_H14C13_ and ^3^*J*_H14C12_ (green, offset below) with the parent CLIP-HSQMBC of the unresolved H11/H14 crosspeaks.

The recently characterized natural product securidine A contains a spin system that has a high degree of chemical “symmetric equivalence” of positions 11–14 in an arginine sidechain spin-system, and thus results in nearly overlapped resonances that are very challenging to access (Fig. 3a).^20^ In this spin system, H11 and H14 are partially overlapping in the proton dimension (Δ*δ* = 6 Hz, multiplet total width = 18.1 Hz), and both protons have long range CH couplings to the same carbon atoms, C12 and C13. The attached protons, H12 and H13, are completely overlapping in the proton dimension (Δ*δ* < 1 Hz). This spin system was used to challenge the CSSF-CLIP-HSQMBC sequence to evaluate the possibility of measuring the proton–carbon couplings individually in a near perfectly overlapped spin system. The CSSF could successfully select clean 6.2 Hz quartets from the partially overlapping H11 and H14 signals (Fig. 3b).

The selection profile was used in a CSSF-CLIP-HSQMBC to produce the individual H11 to C12/C13 and H14 to C12/C13 correlations. In this example the experiment was acquired with very high resolution in F1 without any interference from aliased peaks. These CLIP-HSQMBC resonances would be indistinguishable in an ordinary selective CLIP-HSQMBC (Fig. 3c).

**Fig. 4:**
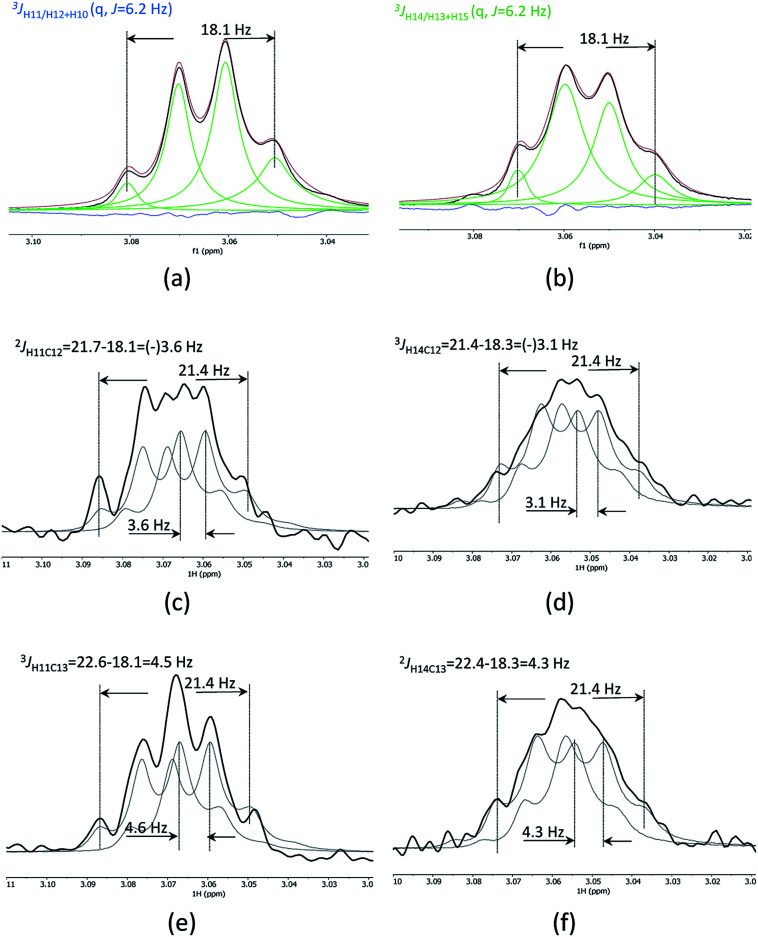
Clean CSSF selected multiplets of H11 (a) and H14 of securidine A, line fitted as quartets with *J* = 6.2 Hz (b). Cross sections through the CSSF-CLIP-HSQMBC crosspeaks for H11/C12 (c), H14/C12 (d), H11/C13 (e) and H14/C13 (f). The CSSF multiplets were used as reference peaks, and two reference multiplets were offset to fit the CLIP-HSQMBC cross-section multiplet. This offset corresponds to the extra splitting caused by the ^*n*^*J*_CH_ coupling.

1D cross-sections through the CSSF-CLIP-HSQMBC peaks were extracted (Fig. 4c–f) and compared to the CSSF 1D reference peaks (Fig. 4a and b). The resulting multiplets were initially too complex to allow direct measurement of the extra CH splitting. The long range ^2^*J*_C12H11_, ^3^*J*_C13H11_, ^2^*J*_C13H14_ and ^3^*J*_C12H14_ coupling constants were therefore measured using two different approaches, both previously described.^5^

The first method uses the sum of couplings, measuring the separation of the two outermost maxima of the multiplets. The reference peak holds the sum of all proton couplings whereas the HSQMBC cross section holds the sum of all proton couplings plus the selected ^*n*^*J*_CH_ coupling. The difference between the two sums yields the heteronuclear coupling constant.

The second method fits two HSQMBC cross sections to the reference peak by offsetting them (Fig. 4c–f), and the offset corresponds to the heteronuclear coupling constant. This method is preferred as it can be difficult to determine the outermost maximums of a multiplet if the signal to noise is low. Furthermore, fitting two multiplets by offsetting them is more robust in the presence of small phase distortions from weak *J*_HH_ coupling or imperfect purging, or in the presence of marginal second order effects.

The long-range heteronuclear proton carbon coupling constants in securidine A were thus successfully determined as follows: ^2^*J*_C12H11_ = 3.6, ^3^*J*_C13H11_ = 4.6, ^2^*J*_C13H14_ = 4.3 and ^3^*J*_C12H14_ = 3.1 Hz.

## Experimentals

### Quinine

NMR experiments were recorded on a 50 mM sample of quinine in DMSO-*d*_6_. The instrument was a Bruker Avance Neo Nanobay spectrometer operating at 400 MHz for protons, equipped with a liquid nitrogen cooled broad-band observe cryoprobe (Prodigy BBO) with cryogenic enhancement for ^1^H, ^2^H and all tunable X nuclei (^15^N–^31^P). Experimental parameters of the CSSF-CLIP-HSQMBC experiment (see Fig. 1): the length of the CSSF increment, *Δ*, was 12.5 ms and a 15.8 ms 180° Gaussian pulse was used as a selective 180° pulse on protons. Adiabatic CHIRP shapes with a sweep width of 60 kHz were used for inversion (0.5 ms) and refocusing (2 ms) 180° carbon pulses. Smoothed square-shaped gradients of 1 ms duration were used, followed by a recovery delay of 200 µs. The gradient amplitude ratios for *G*_1_ : *G*_2_ : *G*_3_ : *G*_4_ : *G*_5_ are 80 : 20.1 : 33 : 50 : 17. Gradient strengths are given as percentages of the absolute gradient strength of approximately 53.5 G cm^−1^. The acquisition times *t*_2_ and *t*_1_ were 0.78 s (spectral width 5263 Hz, 8k complex data points) and 6.36 ms (spectral width 10 060 Hz, 128 real data points), respectively. The relaxation delay was 1.5 s and 2 scans were accumulated for each of the CSSF increments (*n* = 8), resulting in 16 scans per *t*_1_ increment. Zero filling to 512 points in F1, 8096 points in F2 and sine-squared window function in both F1 and F2 dimensions were applied before Fourier transformation of 2D data.

### Securidine A

All NMR experiments were acquired on a sample of 2.0 mg isolated securidine A dissolved in 1 : 1 DMSO-*d*_6_ : chloroform-*d*_1_. The instrument was a Bruker Avance III HD spectrometer operating at 600 MHz for protons, equipped with an inverse detected TCI Helium Cryoprobe with cryogenic enhancement for ^1^H, ^2^H and ^13^C. The experimental parameters of the CSSF-CLIP-HSQMBC were typically: ns = 8, *t*_d0_ = 8, *t*_d2_ = 2k, *t*_d1_ = 64. A 41.6 Hz wide Gaussian pulse was used for selective refocussing of the measured proton and the maximum gradient strength was 65.7 G cm^−1^, otherwise identical settings were used as for quinine above. The data was zero filled to 8k complex points in the direct dimension and forward linear predicted to 1k complex points in the indirect dimension using 2 coefficients, and multiplied with a 45 degrees sine-squared function. The 1D cross section through each peak was then fitted to two reference peaks taken from the CSSF 1D, where the offset determined the ^*n*^*J*_CH_ coupling constant.

## Conclusions

In summary, we present a development that enables the measurement of long-range heteronuclear coupling constants in severely crowded spectral regions by using a chemical shift selective filter as means to eliminate any off-resonance signals in the original CLIP-HSQMBC pulse sequence. We show that an offset of as little as 1–2 Hz is enough to allow for a clean filtration and accurate measurements of coupling constants. The CSSF selection is not dependent on being part of a spin system (like a selective TOCSY for instance), hence the method is generally applicable to any coupling of interest that is not perfectly isochronous with an interfering resonance, or are mutually coupled to each other. We further present a homonulcear decoupled version of the experiment and show that it is possible to reliably measure the heteronuclear coupling constants directly as the splitting in the direct dimension as long as the acquisition blocks are at least twice as long as the selective pulse used in the bshd element.

## Conflicts of interest

There are no conflicts to declare.

## Supplementary Material

RA-009-C9RA04118D-s001
