# Supplementary material for: CSSF-CLIP-HSQMBC: measurement of heteronuclear coupling constants in severely crowded spectral regions
Source: RSC Adv. 2019 Nov 5;9(62):36082–7. doi: 10.1039/c9ra04118d (PMC9074913; doi:10.1039/c9ra04118d)
Supplement: RA-009-C9RA04118D-s001 [file RA-009-C9RA04118D-s001.pdf]

Electronic Supplementary Material (ESI) for RSC Advances.
This journal is © The Royal Society of Chemistry 2019

# CSSF-CLIP-HSQMBC: Measurement of heteronuclear couplings in severely crowded spectral regions

Aitor Moreno,[1] Kine Østnes Hansen[2] and Johan Isaksson*[3]

[1]Bruker BioSpin AG, Application Science department, CH-8117 Fällanden, Switzerland

[2]Marbio, UiT - The Arctic University of Norway, Breivika, NO-9037 Tromsø, Norway

[3]Department of Chemistry, UiT - The Arctic University of Norway, Breivika, NO-9037 Tromsø, Norway

## SUPPORTING INFORMATION

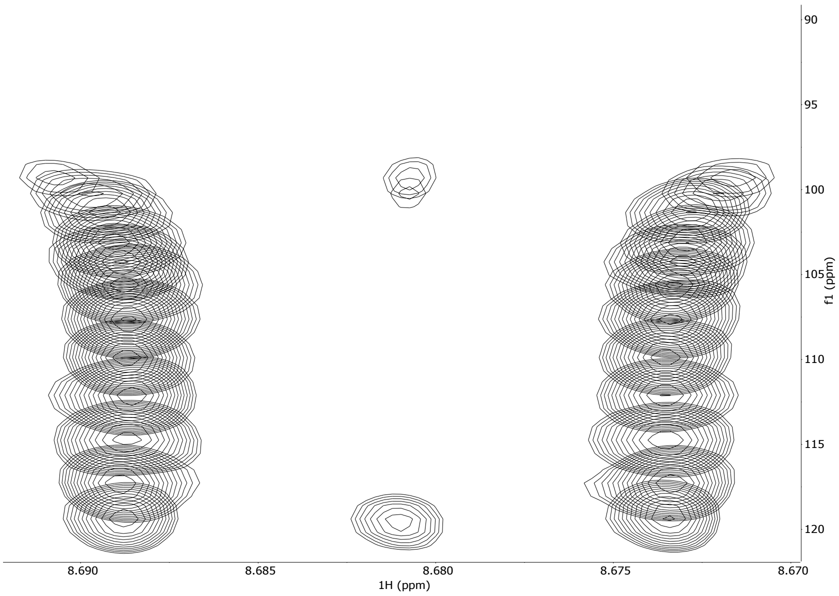


**Figure S1** Stacked plot of a representative crosspeak ($^{2}J_{C3'H2'}$) displaying scaling with diminishing block size (d62, bottom to top). Splittings for this and two more peaks are plotted in Figure S2. Parameters: constant acquisition time = 0.85 s, acquisition block length (# of loops) = 142 (3), 107 (4), 85 (5), 71 (6), 61 (7), 53 (8), 43 (10), 36 (12), 28 (15), 21 (20), 18 (24) ms. Selective pulse duration = 40 ms.

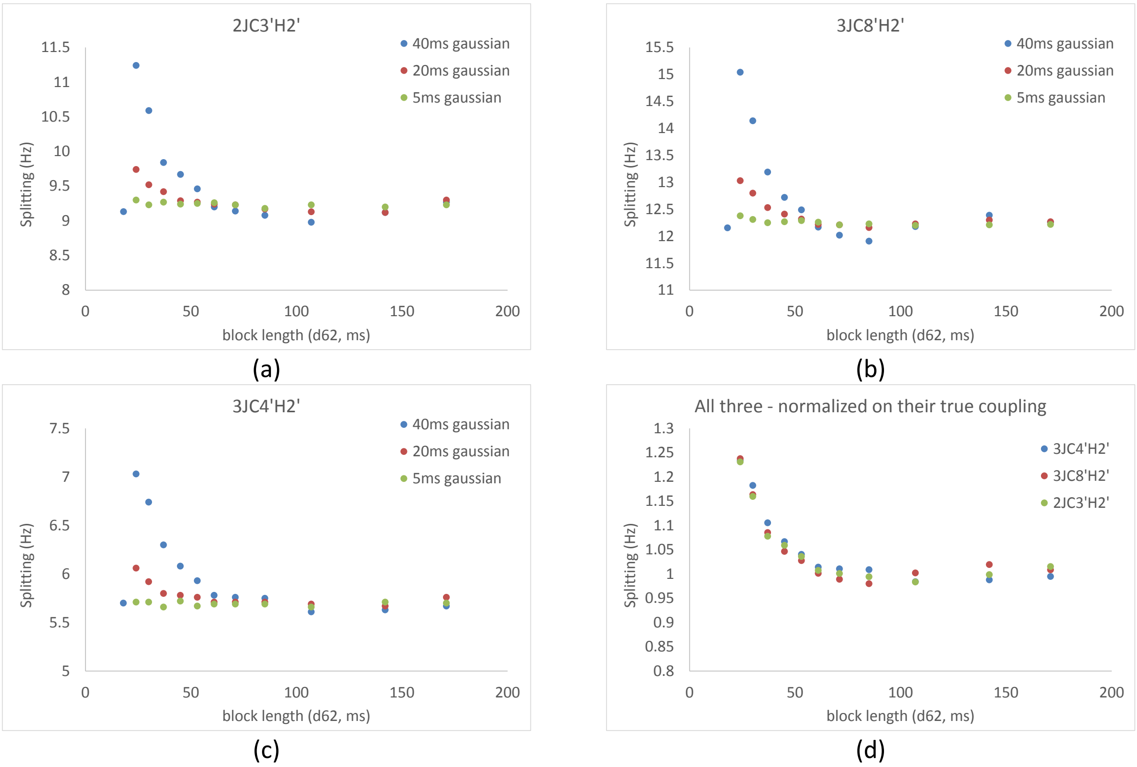


**Figure S2** Measured splitting in the real-time band-selective homodecoupled CLIP-HSQMBC spectrum as a function of the block length and the duration of the selective bshd pulse for

(a) the $^2J_{C3'H2'}$, (b) the $^3J_{C8'H2'}$ and (c) the $^3J_{C4'H2'}$ couplings. (d) All three splittings from the 40ms gaussian series normalized on the true coupling, showing that they scale uniformly. Parameters: constant acquisition time = 0.85 s, acquisition block length (# of loops) = 142 (3), 107 (4), 85 (5), 71 (6), 61 (7), 53 (8), 43 (10), 36 (12), 28 (15), 21 (20) and 18 (24) ms. Selective pulse duration = 5, 20 and 40 ms.

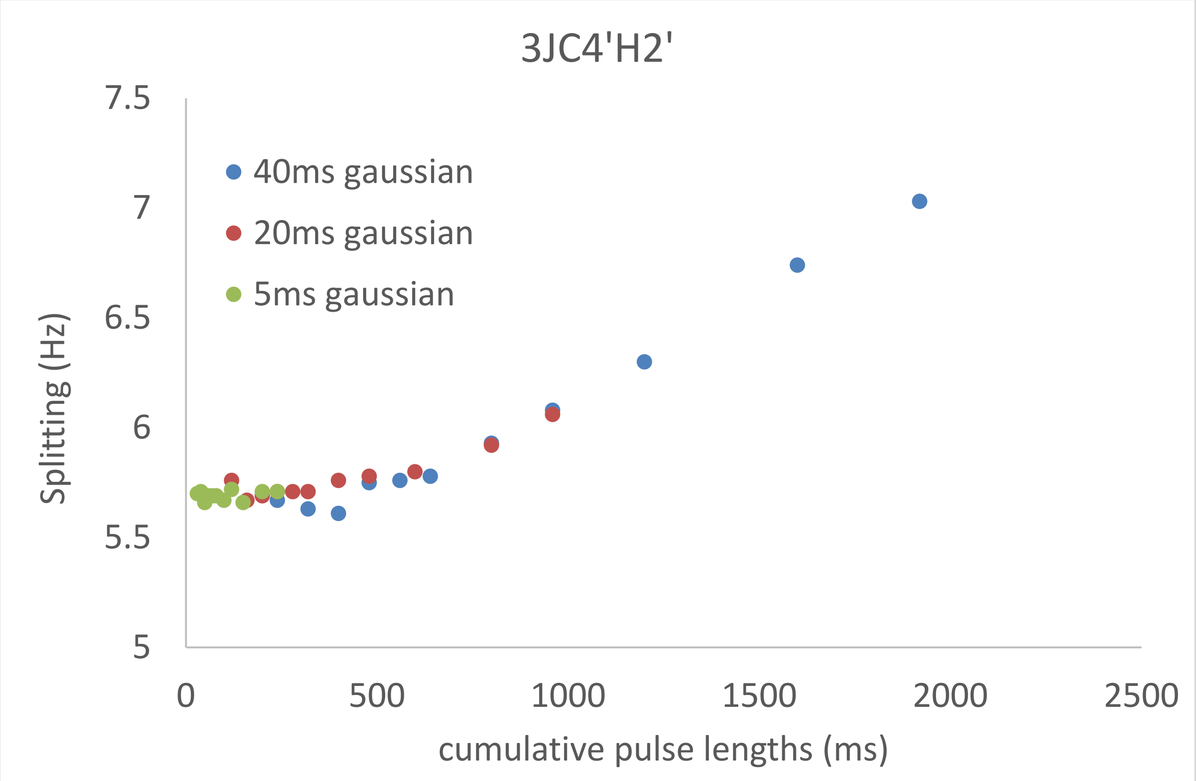


**Figure S3** One representative splitting ($^3J_{C4'H2'}$) as a function of cumulative pulse length of the selective bshd pulses at a constant acquisition time. Parameters: constant acquisition time = 0.85 s, acquisition block length (# of loops) = 142 (3), 107 (4), 85 (5), 71 (6), 61 (7), 53 (8), 43 (10), 36 (12), 28 (15), 21 (20) and 18 (24) ms. Selective pulse duration = 5, 20 and 40 ms.

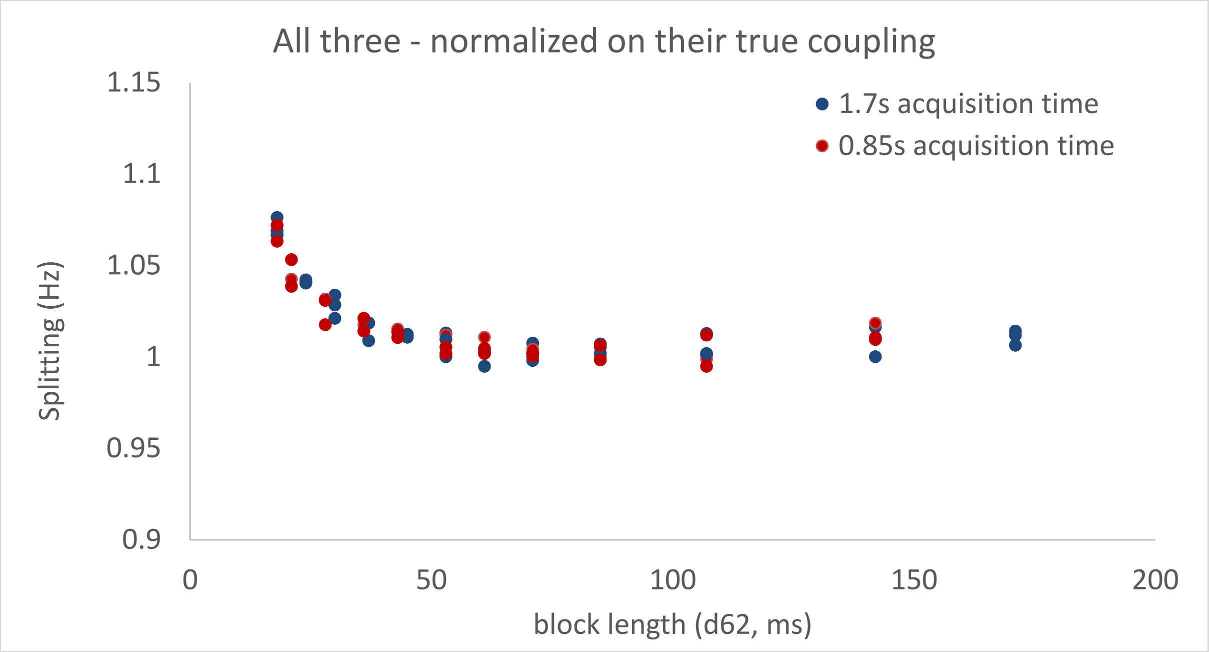


**Figure S4** All three splittings normalized on the true coupling using two different total acquisition times (red: 0.85 s, blue: 1.7 s), showing a dependence relative to the acquisition block size, and not the total number of applied pulses. Parameters: acquisition time = 0.85 and 1.7 s, acquisition block length (# of loops) = 142 (3), 107 (4), 85 (5), 71 (6), 61 (7), 53 (8), 43 (10), 36 (12), 28 (15), 21 (20) and 18 (24) ms for 0.85 s acquisition and doubled number of loops to achieve the same block lengths for 1.7 s acquisition time. Constant selective pulse duration = 20 ms.

# Bruker pulse sequences of the CSSF-CLIP-HSQMBC experiment

**cssf_clip_hsqmbc**

```
;cssf_clip_hsqmbc
;avance-version (12/01/11)
;CSSF-CLIP-HSQMBC
;2D proton-selective CSSF-CLIP-HSQMBC experiment
;using selective refocussing with a shaped pulse
;with chemical shift selective filter (CSSF)
;single echo
;for an esay measurement of long-range proton-carbon coupling constants
;phase sensitive using Echo/Antiecho-TPPI gradient selection
;using shaped pulses for all 180degree pulses on f2 - channel
;
;P.T.Robinson, T.N. Pham & D. Uhrin, J. Magn. Reson. 170, 97-103 (2004)
;(S.J. Duncan, R. Lewis, M.A. Bernstein & P. Sandor, Magn. Reson. Chem. 45, 283-288 (2007))
;J. Saurí, T. Parella, JF. Espinosa, Org. Biomol. Chem. 11, 4473-4478 (2013)
;
;$CLASS=HighRes
;$DIM=2D
;$TYPE=
;$SUBTYPE=
```

```
;$COMMENT=
#include <Avance.incl>
#include <Grad.incl>
#include <Delay.incl>

"p2=p1*2"
"d4=1s/(cnst2*4)"
"d11=30m"

"d0=3u"
"in0=inf1/2"

"DELTA1=p16+d16+50u"
"DELTA2=d4-larger(p12,p14)/2-p16-d16-50u"
"DELTA=p16+d16+50u+p2+d0*2+50u"

"d20=3u"
"in20=1s/(cnst20*td0*2)"
"spoff2=0"

"acqt0=0"
baseopt_echo

1 ze
  d11
2 30m
  20u pl1:f1 BLKGRAD
  d1
  50u UNBLKGRAD
  p1 ph1

  d20
  p16:gp1
  d16
  (center (p12:sp2 ph12) (p14:sp3 ph6):f2 )
  2u
  2u pl1:f1
  (p2 ph1)
  p16:gp1*-1
  d16
  4u
  d20

  50u
  p16:gp3
  d16
  DELTA2
  4u
  (center (p12:sp2 ph1) (p14:sp3 ph6):f2 )
  4u
  DELTA2 pl2:f2 pl1:f1
  50u
  p16:gp3
  d16
  (center (p1 ph2):f1 (p3 ph3):f2 )
  d0
  (p2 ph5)
  d0
  50u
  p16:gp1*EA
  d16
  50u
  (p24:sp7 ph4):f2
   DELTA pl2:f2
  (p3 ph4):f2
  50u
  p16:gp4
  d16
  (p1 ph1):f1
  50u
  p16:gp5
```

```
  d16
  DELTA2
  (center (p12:sp2 ph1) (p14:sp3 ph1):f2 )
  DELTA2 pl2:f2 pl1:f1
  50u
  p16:gp5
  4u
  p16:gp2
  d16 BLKGRAD
  (p3 ph1):f2
  go=2 ph31
  30m mc #0 to 2
    F0(id20 & zd)
    F1EA(exec(rd20) & calgrad(EA), caldel(d0, +in0) & calph(ph3, +180) & calph(ph6, +180) & calph(ph31, +180))

exit

ph1=0
ph2=1
ph3=0 2
ph4=0
ph5=0
ph6=0
ph12=0
ph31=0 2

;pl1 : f1 channel - power level for pulse (default)
;pl2 : f2 channel - power level for pulse (default)
;sp2: f1 channel - shaped pulse
;sp3: f2 channel - shaped pulse (180degree inversion)
;spnam3: Crp60,0.5,20.1
;sp7: f2 channel - shaped pulse (180degree refocussing)
;spnam7: Crp60comp.4
;p1 : f1 channel -  90 degree high power pulse
;p2 : f1 channel - 180 degree high power pulse
;p3 : f2 channel -  90 degree high power pulse
;p12: f1 channel - 180 degree shaped pulse
;p14: f2 channel - 180 degree shaped pulse for inversion
;     = 500usec for Crp60,0.5,20.1
;p16: homospoil/gradient pulse                       [1 msec]
;p24: f2 channel - 180 degree shaped pulse for refocussing
;     = 2msec for Crp60comp.4
;d0 : incremented delay (2D)                         [3 usec]
;d1 : relaxation delay; 1-5 * T1
;d4 : 1/(4J)XH
;d11: delay for disk I/O                             [30 msec]
;d16: delay for homospoil/gradient recovery
;d20: incremented delay                              [3 usec]
;cnst2: = J(XH)
;cnst20: distance (in Hz) to next multiplet (to be suppressed)
;in20: 1s/(cnst20*td0*2)
;inf1: 1/SW(X) = 2 * DW(X)
;in0: 1/(2 * SW(X)) = DW(X)
;nd0: 2
;ns: 2 * n, total number of scans: NS * TD0
;ds: >= 16
;td0: TD0 = number of steps for suppression of undesired signals [8-16]
;td1: number of experiments
;FnMODE: echo-antiecho

;use gradient ratio:  gp 1 : gp 2 : gp 3 : gp 4 : gpz5
;                      80 : 20.1 :   33 :   50 :   17         for C-13

;for z-only gradients:
;gpz1: 80%
;gpz2: 20.1% for C-13
;gpz3: 33%
;gpz4: 50%
;gpz5: 17%

;use gradient files:
```

```
;gpnam1: SMSQ10.100
;gpnam2: SMSQ10.100
;gpnam3: SMSQ10.100
;gpnam4: SMSQ10.100
;gpnam5: SMSQ10.100

;$Id: hsqcetgpsisp2.2,v 1.8 2012/01/31 17:49:26 - Exp $
```

**cssf_clip_hsqmbc_bshd (Avance NEO version)**

```
;cssf_clip_hsqmbc_bshd_NEO
;avance-version (12/01/11)
;CSSF-CLIP-HSQMBC
;2D proton-selective CSSF-CLIP-HSQMBC experiment
;using selective refocussing with a shaped pulse
;with chemical shift selective filter (CSSF)
;single echo
;for an esay measurement of long-range proton-carbon coupling constants
;phase sensitive using Echo/Antiecho-TPPI gradient selection
;using shaped pulses for all 180degree pulses on f2 - channel
;with bandselective homodecoupling
;
;P.T.Robinson, T.N. Pham & D. Uhrin, J. Magn. Reson. 170, 97-103 (2004)
;(S.J. Duncan, R. Lewis, M.A. Bernstein & P. Sandor, Magn. Reson. Chem. 45, 283-288 (2007))
;J. Saurí, T. Parella, JF. Espinosa, Org. Biomol. Chem. 11, 4473-4478 (2013)
;L. Castanar, P. Nolis A. Virgili & T. Parella, Chem. Eur. J. 19, 17283-17286 (2013)
;J. Ying, J. Roche & A. Bax, J. Magn. Reson. 241, 97-102 (2014)
;
;$CLASS=HighRes
;$DIM=2D
;$TYPE=
;$SUBTYPE=
;$COMMENT=


#include <Avance.incl>
#include <Grad.incl>
#include <Delay.incl>
#include <De.incl>


"p2=p1*2"
"d4=1s/(cnst2*4)"
"d11=30m"

"p29=300u"

"d0=3u"
"in0=inf1/2"


"DELTA1=p16+d16+50u"
"DELTA2=d4-larger(p12,p14)/2-p16-d16-50u"
"DELTA=p16+d16+50u+p2+d0*2+50u"

"d20=3u"
"in20=1s/(cnst20*td0*2)"

"spoff2=0"

"d62=aq/(l0*2)"
"d63=d62/2"

"acqt0=0"
baseopt_echo


1 ze
  d11
2 30m
  20u pl1:f1 BLKGRAD
```

```
 d1
 50u UNBLKGRAD
 p1 ph1

 d20
 p16:gp1
 d16
 (center (p12:sp2 ph12) (p14:sp3 ph6):f2 )
 2u
 2u pl1:f1
 (p2 ph1)
 p16:gp1*-1
 d16
 4u
 d20

 50u
 p16:gp3
 d16
 DELTA2
 4u
 (center (p12:sp2 ph1) (p14:sp3 ph6):f2 )
 4u
 DELTA2 pl2:f2 pl1:f1
 50u
 p16:gp3
 d16
 (center (p1 ph2):f1 (p3 ph3):f2 )
 d0
 (p2 ph5)
 d0
 50u
 p16:gp1*EA
 d16
 50u
 (p24:sp7 ph4):f2
  DELTA pl2:f2
 (p3 ph4):f2
 50u
 p16:gp4
 d16
 (p1 ph1):f1
 50u
 p16:gp5
 d16
 DELTA2
 (center (p12:sp2 ph1) (p14:sp3 ph1):f2 )
 DELTA2 pl2:f2 pl1:f1
 50u
 p16:gp5
 4u
 p16:gp2
 d16
 (p3 ph1):f2

 10u
 ACQ_START(ph30,ph31)
 0.1u START_NEXT_SCAN
 0.1u REC_UNBLK
 0.05u DWELL_RELEASE
 d63
 0.05u DWELL_HOLD
 0.1u REC_BLK

4 10u
 p29:gp6
 d16 pl1:f1
 (p2 ph7):f1
 p29:gp6
 d16
 10u
```

```
  p29:gp7
  d16
  5u
  (p12:sp2 ph8):f1
  5u
  p29:gp7
  d16

  0.1u REC_UNBLK
  0.05u DWELL_RELEASE
  d62
  0.05u DWELL_HOLD
  0.1u REC_BLK

  10u
  p29:gp6
  d16 pl1:f1
  (p2 ph7):f1
  p29:gp6
  d16
  10u

  p29:gp7
  d16
  5u
  (p12:sp2 ph8):f1
  5u
  p29:gp7
  d16

  0.1u REC_UNBLK
  0.05u DWELL_RELEASE
  d62
  0.05u DWELL_HOLD
  0.1u REC_BLK

  lo to 4 times l0

  d62

  rcyc=2

  30m mc #0 to 2
    F0(id20 & zd)
    F1EA(exec(rd20) & calgrad(EA), caldel(d0, +in0) & calph(ph3, +180) & calph(ph6, +180) & calph(ph31, +180))

  4u BLKGRAD
exit

ph1=0
ph2=1
ph3=0 2
ph4=0
ph5=0
ph6=0
ph7=0
ph8=2
ph12=0
ph30=0
ph31=0 2


;pl1 : f1 channel - power level for pulse (default)
;pl2 : f2 channel - power level for pulse (default)
;sp2: f1 channel - shaped pulse
;sp3: f2 channel - shaped pulse (180degree inversion)
;spnam3: Crp60,0.5,20.1
;sp7: f2 channel - shaped pulse (180degree refocussing)
;spnam7: Crp60comp.4
;p1 : f1 channel -  90 degree high power pulse
```

```
;p2 : f1 channel - 180 degree high power pulse
;p3 : f2 channel -  90 degree high power pulse
;p12: f1 channel - 180 degree shaped pulse
;p14: f2 channel - 180 degree shaped pulse for inversion
;     = 500usec for Crp60,0.5,20.1
;p16: homospoil/gradient pulse                       [1 msec]
;p24: f2 channel - 180 degree shaped pulse for refocussing
;     = 2msec for Crp60comp.4
;p29: gradient pulse 3                               [300 usec]
;d0 : incremented delay (2D)                         [3 usec]
;d1 : relaxation delay; 1-5 * T1
;d4 : 1/(4J)XH
;d11: delay for disk I/O                             [30 msec]
;d16: delay for homospoil/gradient recovery
;d20: incremented delay                              [3 usec]
;cnst2: = J(XH)
;cnst20: distance (in Hz) to next multiplet (to be suppressed)
;in20: 1s/(cnst20*td0*2)
;inf1: 1/SW(X) = 2 * DW(X)
;in0: 1/(2 * SW(X)) = DW(X)
;nd0: 2
;ns: 2 * n, total number of scans: NS * TD0
;ds: >= 16
;td0: TD0 = number of steps for suppression of undesired signals [8-16]
;td1: number of experiments
;FnMODE: echo-antiecho

;use gradient ratio:  gp 1 : gp 2 : gp 3 : gp 4 : gpz5 : gpz6 : gpz7
;                     80 : 20.1 :   33 :   50 :   17 :   3  : 5 for C-13

;for z-only gradients:
;gpz1: 80%
;gpz2: 20.1% for C-13
;gpz3: 33%
;gpz4: 50%
;gpz5: 17%
;gpz6: 3%
;gpz7: 5%

;use gradient files:
;gpnam1: SMSQ10.100
;gpnam2: SMSQ10.100
;gpnam3: SMSQ10.100
;gpnam4: SMSQ10.100
;gpnam5: SMSQ10.100
;gpnam6: SMSQ10.100
;gpnam7: SMSQ10.100

;$Id: hsqcetgpsisp2.2,v 1.8 2012/01/31 17:49:26 - Exp $
```

## cssf_clip_hsqmbc_bshd (Avance III version)

```
;cssf_clip_hsqmbc_bshd_AViii
;avance-version (12/01/11)
;CSSF-CLIP-HSQMBC
;2D proton-selective CSSF-CLIP-HSQMBC experiment
;using selective refocussing with a shaped pulse
;with chemical shift selective filter (CSSF)
;single echo
;for an esay measurement of long-range proton-carbon coupling constants
;phase sensitive using Echo/Antiecho-TPPI gradient selection
;using shaped pulses for all 180degree pulses on f2 - channel
;with bandselective homodecoupling
;
;P.T.Robinson, T.N. Pham & D. Uhrin, J. Magn. Reson. 170, 97-103 (2004)
;(S.J. Duncan, R. Lewis, M.A. Bernstein & P. Sandor, Magn. Reson. Chem. 45, 283-288 (2007))
;J. Saurí, T. Parella, JF. Espinosa, Org. Biomol. Chem. 11, 4473-4478 (2013)
;L. Castanar, P. Nolis A. Virgili & T. Parella, Chem. Eur. J. 19, 17283-17286 (2013)
;J. Ying, J. Roche & A. Bax, J. Magn. Reson. 241, 97-102 (2014)
```

```
;
;$CLASS=HighRes
;$DIM=2D
;$TYPE=
;$SUBTYPE=
;$COMMENT=


#include <Avance.incl>
#include <Grad.incl>
#include <Delay.incl>
#include <De.incl>


"p2=p1*2"
"d4=1s/(cnst2*4)"
"d11=30m"

"p29=300u"

"d0=3u"
"in0=inf1/2"


"DELTA1=p16+d16+50u"
"DELTA2=d4-larger(p12,p14)/2-p16-d16-50u"
"DELTA=p16+d16+50u+p2+d0*2+50u"

"d20=3u"
"in20=1s/(cnst20*td0*2)"

"spoff2=0"

"d62=aq/(l0*2)"
"d63=d62/2"

"acqt0=0"
baseopt_echo
dwellmode explicit


1 ze
  d11
2 30m
  20u pl1:f1 BLKGRAD
  d1
  50u UNBLKGRAD
  p1 ph1

  d20
  p16:gp1
  d16
  (center (p12:sp2 ph12) (p14:sp3 ph6):f2 )
  2u
  2u pl1:f1
  (p2 ph1)
  p16:gp1*-1
  d16
  4u
  d20

  50u
  p16:gp3
  d16
  DELTA2
  4u
  (center (p12:sp2 ph1) (p14:sp3 ph6):f2 )
  4u
  DELTA2 pl2:f2 pl1:f1
  50u
  p16:gp3
```

```
 d16
 (center (p1 ph2):f1 (p3 ph3):f2 )
 d0
 (p2 ph5)
 d0
 50u
 p16:gp1*EA
 d16
 50u
 (p24:sp7 ph4):f2
  DELTA pl2:f2
 (p3 ph4):f2
 50u
 p16:gp4
 d16
 (p1 ph1):f1
 50u
 p16:gp5
 d16
 DELTA2
 (center (p12:sp2 ph1) (p14:sp3 ph1):f2 )
 DELTA2 pl2:f2 pl1:f1
 50u
 p16:gp5
 4u
 p16:gp2
 d16
 (p3 ph1):f2

 10u
 ACQ_START(ph30,ph31)
 0.1u REC_UNBLK
 0.05u DWL_CLK_ON
 d63
 0.05u DWL_CLK_OFF
 0.1u REC_BLK

4 10u
 p29:gp6
 d16 pl1:f1
 (p2 ph7):f1
 p29:gp6
 d16
 10u

 p29:gp7
 d16
 5u
 (p12:sp2 ph8):f1
 5u
 p29:gp7
 d16

 0.1u REC_UNBLK
 0.05u DWL_CLK_ON
 d62
 0.05u DWL_CLK_OFF
 0.1u REC_BLK

 10u
 p29:gp6
 d16 pl1:f1
 (p2 ph7):f1
 p29:gp6
 d16
 10u

 p29:gp7
 d16
 5u
 (p12:sp2 ph8):f1
```

```
  5u
  p29:gp7
  d16

  0.1u REC_UNBLK
  0.05u DWL_CLK_ON
  d62
  0.05u DWL_CLK_OFF
  0.1u REC_BLK

  lo to 4 times l0

  d62

  rcyc=2

  30m mc #0 to 2
    F0(id20 & zd)
    F1EA(exec(rd20) & calgrad(EA), caldel(d0, +in0) & calph(ph3, +180) & calph(ph6, +180) & calph(ph31, +180))

  4u BLKGRAD
exit

ph1=0
ph2=1
ph3=0 2
ph4=0
ph5=0
ph6=0
ph7=0
ph8=2
ph12=0
ph30=0
ph31=0 2


;pl1 : f1 channel - power level for pulse (default)
;pl2 : f2 channel - power level for pulse (default)
;sp2: f1 channel - shaped pulse
;sp3: f2 channel - shaped pulse (180degree inversion)
;spnam3: Crp60,0.5,20.1
;sp7: f2 channel - shaped pulse (180degree refocussing)
;spnam7: Crp60comp.4
;p1 : f1 channel -  90 degree high power pulse
;p2 : f1 channel - 180 degree high power pulse
;p3 : f2 channel -  90 degree high power pulse
;p12: f1 channel - 180 degree shaped pulse
;p14: f2 channel - 180 degree shaped pulse for inversion
;     = 500usec for Crp60,0.5,20.1
;p16: homospoil/gradient pulse                       [1 msec]
;p24: f2 channel - 180 degree shaped pulse for refocussing
;     = 2msec for Crp60comp.4
;p29: gradient pulse 3                               [300 usec]
;d0 : incremented delay (2D)                         [3 usec]
;d1 : relaxation delay; 1-5 * T1
;d4 : 1/(4J)XH
;d11: delay for disk I/O                             [30 msec]
;d16: delay for homospoil/gradient recovery
;d20: incremented delay                              [3 usec]
;cnst2: = J(XH)
;cnst20: distance (in Hz) to next multiplet (to be suppressed)
;in20: 1s/(cnst20*td0*2)
;inf1: 1/SW(X) = 2 * DW(X)
;in0: 1/(2 * SW(X)) = DW(X)
;nd0: 2
;ns: 2 * n, total number of scans: NS * TD0
;ds: >= 16
;td0: TD0 = number of steps for suppression of undesired signals [8-16]
;td1: number of experiments
;FnMODE: echo-antiecho
```

;use gradient ratio:  gp 1 : gp 2 : gp 3 : gp 4 : gpz5 : gpz6 : gpz7
;                     80 : 20.1 :   33 :   50 :   17 :   3  : 5 for C-13

;for z-only gradients:
;gpz1: 80%
;gpz2: 20.1% for C-13
;gpz3: 33%
;gpz4: 50%
;gpz5: 17%
;gpz6: 3%
;gpz7: 5%

;use gradient files:
;gpnam1: SMSQ10.100
;gpnam2: SMSQ10.100
;gpnam3: SMSQ10.100
;gpnam4: SMSQ10.100
;gpnam5: SMSQ10.100
;gpnam6: SMSQ10.100
;gpnam7: SMSQ10.100

;$Id: hsqcetgpsisp2.2,v 1.8 2012/01/31 17:49:26 - Exp $
